# Progress in Medicine

**Published:** 1898-11-26

**Authors:** 


### Progress in Medicine.

### CIRCULATORY SYSTEM.

**Aneurysm.**—Renn[1] regards the five following symptoms as diagnostic of aneurysm: A pulsating tumour in the course of a vessel, murmur, inequality of pulses, pressure symptoms, and pain. Aneurysms of the descending thoracic aorta, of the abdominal aorta, and vessels of the brain present the greatest difficulties of diagnosis. The pleura, lung, and œsophagus are capable of undergoing considerable pressure without giving rise to symptoms, and the nerves of the trunk are well protected. Aneurysm of the descending thoracic aorta may long remain unsuspected. Details of a fatal case occurring in the German Hospital of New York are given, together with a post-mortem report. The aorta was atheromatous throughout, and the kidneys showed syphilitic changes. The stages in this case appear to have been: Firstly, a complete and sharply-defined perforation of the vascular wall, the sac wall being formed by the matting together of the adventitia with the pleura and lung; later this primary sac ruptured into the posterior mediastinum, and some bleeding took place into the left pleural cavity, leading to compression of the lung and acute anæmia. Death was due to a further extension of the hæmorrhage. During life there was little pain and no dysphagia. New growth in the left lung was suspected, but puncture of the pleural cavity, which furnished pure blood, led to correct diagnosis.

Bacelli[2] gives a clinical description of four cases of aneurysm of the aorta. One, in a youth of seventeen, was in the ascending part of the arch. It presented no external bulging, and there were two well-marked bruits to be heard. In the others there was distinct bulging of the chest wall; two were situated in the ascending part of the arch, and one in the thoracic aorta opposite the tenth dorsal vertebra. This last case differed from the other two in that there was only one bruit audible, whilst in the others there were two. In the first case the aortic valves were incompetent, and the left ventricle hypertrophied. In the other three cases there was no alteration in the heart. Bacelli then considers what factors determine the hypertrophic change in the heart-wall, and what is the true explanation of the double bruit. He finds that hypertrophy, or dilatation of the ventricle wall, is not necessarily, nor indeed frequently, associated with aortic aneurysm. Sacculated aneurysms with narrowed orifices produce no notable deviation of the central hydraulic axis of the vessel, and so do not accentuate the intracardiac pressure. Aneurysms with a wider neck, such as the cylindrical and fusiform varieties, cause a marked deviation of this axis, and are thus associated with dilatation and compensatory hypertrophy.

The second bruit heard over aneurysmal sacs situated in the arch of the aorta he regards as due to the transmission of the aortic second sound, and in no way connected with any contraction of the walls of the aneurysm itself. A second diastolic pulsation which may sometimes be detected in the sac by auscultation, palpation, or by the sphygmograph, takes place when the neck of the sac is wide, the blood pressure high, and the arterial walls moderately elastic. Bassen[3] relates a case of aneurysm of the abdominal aorta just below the cœliac axis, on the posterior surface. It was discovered post-mortem, and had apparently given rise to no symptoms during life. The heart was dilated and fatty, with calcareous plaques upon the aortic valves. The vessels were generally atheromatous. The right femoral and popliteal arteries were the seat of a large aneurysm, and there was a dilatation about the size of a hen's egg in the left popliteal. The carotids were thinned and dilated, and where they connect in the skull to form the circle of Willis, there was a dilatation about the size of a hazel-nut. The patient died of broncho-pneumonia. The sudden development and rapid enlargement of an aneurysmal sac is well illustrated by a case reported by Martin.[4] A seaman, whilst blowing his boatswain's whistle, felt something

"give" in his neck. On examination there was found to be an aneurysm of the left external carotid just above the point it was given off. There was some loss of voice, cough, feeble and irregular heart's action, and contraction of the left pupil. Eleven days from the commencement, the tumour had increased to the size of a hen's egg. The left common carotid was ligatured above the omohyoid, and three weeks later the patient was in normal health, the sac hard, decreased in size, the voice thin and boyish, and the left pupil still somewhat contracted.

A case of complete rupture of the aorta, in which life was prolonged for more than four hours, is reported by Ashwin.[5] The patient, a homeless tramp, fell suddenly in the street. He was unconscious and collapsed. The pulse could just be felt at the wrist, the respirations were infrequent and stertorous. Pupils dilated. No reflexes. Cerebral hæmorrhage was diagnosed. Death occurred four and a half hours after the fall. Post-mortem examination showed the pericardium to contain six ounces of blood. There was hypertrophy of the left ventricle, no valvular lesion, and a rupture of the aorta about three-quarters of an inch above the aortic valves. The blood clot lay between the pericardium and the outer wall of the aorta as high as the origin of the innominate artery. The ascending portion of the aorta was invaginated into the transverse portion, and beyond this there was yet a second invagination. The ruptured end of the aorta had been turned inwards and passed through a rent in the arterial coats just below the origin of the innominate artery. Blood was thus shut off from the transverse aorta. A second invagination had resulted in the ruptured end again turning in and passing through a second rent in the arterial walls into the lumen of the transverse aorta. This double invagination explains how the lumen of the transverse arch was restored, so that the circulation was maintained and life prolonged for so long a time.

Newman[6] states that the earliest indications of intrathoracic aneurysms of the aorta or innominate are to be obtained by seeking for signs of pressure upon the vagus and recurrent laryngeal nerves. Sudden and paroxysmal dyspnœa accompanied by laryngeal stridor, is one of the earliest symptoms. During the intervals the laryngoscope may show no alteration in the larynx, but as the case progresses some paresis always develops. When the spasm or paralysis of the vocal cord is limited to one side the probability is that the recurrent laryngeal on that side is alone compressed, and the danger arising from the dyspnœa need not cause alarm. If paralysis or spasm is bilateral either the trunk of one vagus, or of both recurrents, are pressed upon, a much more serious condition. Cough caused by pressure on nerve fibres supplying the larynx is characterised by being hoarse and imperfect, noisy and brassy in tone. The voice is produced with some little difficulty, and phonation is imperfect. A case of dissecting aneurysm[7] is described by Coleman. The patient was a house painter, aged 65. There was pain in the back and loss of power in the legs, both of sudden onset. Arteries were atheromatous, heart hypertrophied, pulse high tensioned. There were no signs of aneurysm during life, and pulsation could be felt in both femoral arteries. No affection of sphincters of bladder and rectum. Knee jerks and plantar reflexes lost. On the day following the onset the pain and paralysis disappeared, but death was sudden 52 hours after the first symptoms. At the post-mortem the right pleural cavity was filled with blood, the arch and upper part of the descending aorta were distended, and formed a tumour-like mass from which blood had escaped into the right pleura. The large vessels were markedly atheromatous, and a dissecting aneurysm in the coats of the aorta and iliac arteries extended from the innominate artery to the left femoral artery about an inch below Poupart's ligament. In the aorta the coats were separated by clot for nearly the whole circumference of the vessel. In the smaller arteries it did not extend more than half way around the circumference. The clot lay chiefly in the middle tunic. The position of the internal rupture could not be ascertained. The paraplegia was, in Coleman's opinion, due to interference with the arterial supply of the lumbar enlargement of the cord, the pain to the primary rupture and separation of the coats. The determining cause of death was the rupture of the external wall and consequent bleeding into the pleura.

[1] Med. Rec., Sept. 10, 1898. [2] La Semaine Médicale, Mar. 30, 1898. [3] Jour. des Sci. Med. de Lille, June, 1898. [4] Lancet, July 9, 1898. [5] Lancet, July 9, 1898. [6] Glas. Med. Jour., Aug., 1898. [7] Dub. Jour. Med. Sci., Aug., 1898.

